# Scanxiety among Adults with Cancer: A Scoping Review to Guide Research and Interventions

**DOI:** 10.3390/cancers15051381

**Published:** 2023-02-22

**Authors:** Heather M. Derry-Vick, Lauren C. Heathcote, Nina Glesby, Judy Stribling, Matthew Luebke, Andrew S. Epstein, Holly G. Prigerson

**Affiliations:** 1 Center for Discovery and Innovation, Hackensack Meridian Health, Nutley, NJ 07110, USA; 2 Hackensack Meridian School of Medicine, Nutley, NJ 07110, USA; 3 Health Psychology Section, Department of Psychology, Institute of Psychiatry, Psychology and Neuroscience, King’s College London, London SE1 9RT, UK; 4 Smith College, Northampton, MA 01063, USA; 5 Weill Cornell Medicine, New York, NY 10021, USA; 6 Memorial Sloan Kettering Cancer Center, New York, NY 10065, USA

**Keywords:** Scanxiety, cancer, imaging, scan, stress, anxiety

## Abstract

**Simple Summary:**

“Scanxiety”, or the distress and/or anxiety occurring before, during, and after cancer-related imaging/scans, is an upsetting experience during and following cancer. To better understand the nature of scanxiety, related research gaps and practices, and possible ways to help manage it, we conducted a review of the literature using a structured search process. We identified and synthesized findings from 36 articles on scanxiety among adults diagnosed with current or prior cancer. We found that scanxiety occurs across the cancer continuum. The articles also indicated that there are various components of the scan experience that prompt anxiety, such as those related to scan procedures and those related to the implications of important test results. The waiting period between the scan procedure and receipt of the results was described as particularly stressful. Our review also summarizes measures and methods used in scanxiety research. We discuss how the findings of this review may be used to inform future research directions and to generate approaches for helping people to manage scanxiety.

**Abstract:**

**Background**: Scan-related anxiety (“scanxiety”) is distressing to people living with and beyond cancer. We conducted a scoping review to promote conceptual clarity, identify research practices and gaps, and guide intervention strategies for adults with a current or prior cancer diagnosis. **Methods:** Following a systematic search, we screened 6820 titles and abstracts, evaluated 152 full-text articles, and selected 36 articles. Definitions, study designs, measurement methods, correlates, and consequences of scanxiety were extracted and summarized. **Results:** The reviewed articles included individuals living with current cancer (n = 17) and those in the post-treatment phase (n = 19), across a breadth of cancer types and disease stages. In five articles, authors explicitly defined scanxiety. Multiple components of scanxiety were described, including those related to scan procedures (e.g., claustrophobia, physical discomfort) and scan results (e.g., implications for disease status and treatment), suggesting varied intervention approaches may be needed. Twenty-two articles used quantitative methods, nine used qualitative methods, and five used mixed methods. In 17 articles, symptom measures specifically referenced cancer scans; 24 included general measures without reference to scans. Scanxiety tended to be higher among those with lower education levels, less time since diagnosis, and greater baseline anxiety levels (three articles each). Although scanxiety often decreased immediately pre- to post-scan (six articles), participants reported the waiting period between scan and results to be particularly stressful (six articles). Consequences of scanxiety included poorer quality of life and somatic symptoms. Scanxiety promoted follow-up care for some patients yet hindered it for others. **Conclusions:** Scanxiety is multi-faceted, heightened during the pre-scan and scan-to-results waiting periods, and associated with clinically meaningful outcomes. We discuss how these findings can inform future research directions and intervention approaches.

## 1. Introduction

“Scanxiety” (defined here as distress and/or anxiety occurring before, during, and after cancer-related imaging/scans [1,2,3]) is widely recognized as upsetting in patient reports, popular press, and patient education materials [1,4,5]. Given that scans yield highly personal and consequential results regarding disease status, treatment response, and recurrence, it is not surprising that awaiting these results can be anxiety-provoking for patients during and following cancer treatment. Indeed, stress and anxiety are often ranked as highly concerning for patients awaiting scan results [6], and people living with cancer report that scanxiety is a notable and challenging part of their cancer experience [7]. Accordingly, summarizing the emerging empirical research on scanxiety among people with cancer has the potential to advance the science of cancer survivorship, as well as to inform potential intervention approaches that may ease this difficult time period that arises repeatedly for cancer survivors.

In a prior scoping review, Bui and colleagues summarized the quantitative measurement methods, prevalence, and severity of scanxiety among those with and without cancer. They concluded that the literature lacked a consistent definition of scanxiety [8]. There remains a need to clarify scanxiety’s definition and components in order to inform measurement and intervention strategies. A useful approach for doing so is to integrate qualitative research on patients’ experiences. Furthermore, because those with cancer repeatedly experience uncertainty about disease status while undergoing follow-up scans to establish stage and treatment plans, evaluate progression and treatment response, and detect recurrence, they may have unique scanxiety experiences and needs compared to those without cancer who undergo screening procedures. Accordingly, there is a need to advance the conceptualization of scanxiety by including qualitative findings, to review scanxiety literature specifically in those diagnosed with cancer, and to inform broad research directions in this area.

In this systematic scoping review, we synthesized findings from empirical articles on scanxiety among adults living with (e.g., those with current disease or in active treatment) and beyond (e.g., those deemed cured, post-treatment, or in remission) cancer. The objectives of this review were to describe the definitions, measurement methods, symptom levels, and correlates of scanxiety, and to identify research gaps and promising strategies for intervention. To achieve these objectives, we conducted a scoping review with a systematic search strategy. Given the current lack of conceptual clarity and to take an inclusive approach, we considered a range of psychological symptoms around a scan (including distress, stress, and anxiety) to reflect scanxiety. In this review, we describe the multi-faceted nature of scanxiety, outline measures that specifically focus on anxiety with respect to scans versus those that measure more general anxiety, and summarize research conducted to date regarding scanxiety’s correlates and consequences. We then discuss how this information can inform future research directions and opportunities for intervention strategies during this stressful yet under-addressed period in clinical care.

## 2. Methods

Because our goals were to examine and clarify definitions of scanxiety and its components, the methods used to study it, and current knowledge gaps, we selected a scoping review method (vs. a systematic review method to address a more specific research question, [9]). We used a previously-established scoping review methodology to guide our study methods and applied the Preferred Reporting Items for Systematic Reviews and Meta-Analyses for Scoping Reviews [9,10,11]. A protocol describing the objectives, inclusion criteria, and methods was posted on Open Science Framework (osf.io/wm79v) in March 2021 [12]. As described below, the approach was updated to include an interim search and updated data extraction procedures, due to the time elapsed after the updated search as well as institution changes among multiple co-authors. For similar reasons, the data extraction phase included one rater (rather than two as initially planned) to chart the data for each included article.

References were compiled through a systematic search conducted by a librarian with expertise in clinical medicine (J.S.). Based upon initial search terms generated by the corresponding author (H.M.D.V.: cancer; scan; imaging; CT; MRI; recurrence; anxiety; distress; stress; scanxiety), the librarian constructed an extensive search strategy with expanded terms. The three main search clusters were cancer survivors, check-ups involving imaging, and patient anxiety. Medical Subject Headings, Emtree, PsycINFO and Cochrane terms were combined within each cluster using ‘OR’; these clusters were then combined using ‘AND’. The search strategy is available in the Supplementary Materials.

The databases searched were MEDLINE, Embase, PsycINFO, CINAHL, Cochrane and Web of Science. The initial search was conducted in July 2020 for articles published in English between 1 January 1980 and 31 July 2020, and an updated search was conducted in March 2021. In order to include interim articles, the first author also conducted a limited search in PubMed up to July 2022. Reference management, abstract/title and full-text screening decisions, and data extraction utilized the Covidence reference management system.

Prior to initial article screening, references were downloaded into Endnote and then transferred to the Covidence review management system, which was used to identify and remove duplicates. The remaining titles and abstracts were then screened for inclusion using initial eligibility criteria that were clarified through consensus meetings. Titles and abstracts were indicated for inclusion if they reported data from adults undergoing cancer-related imaging tests, who also had a measure of psychological symptoms or recalled symptoms experienced around the time of the scan. Using these criteria, abstracts and titles were screened independently by two team members (N.G., M.L., and/or H.M.D.V.) for possible inclusion. Disagreements for initial screening were resolved via tiebreaker vote from the third member.

For records that met the initial criteria, full texts were reviewed by two study authors (H.M.D.V. and L.C.H.) and evaluated for final inclusion. The initial eligibility criteria above were refined to guide the full-text review phase and revised iteratively via consensus discussions (Table 1). Inclusion and exclusion decisions (along with exclusion reasons) for the full-text review phase were recorded using Covidence. Disagreements were resolved via consensus discussion between the two raters.

The first author developed a data abstraction template and utilized this tool to extract and chart the data from the included articles. The following information was extracted by a single rater from the included articles as available: study design and methodology; demographic and cancer-related characteristics of participants; study definition of scanxiety; methods, instrument, and timing of scanxiety assessment; levels of scanxiety symptoms from quantitative measures; predictors and correlates of scanxiety; characteristics of the scan; and descriptions and effects of interventions. For qualitative articles, we also extracted themes and descriptions of scanxiety. Based on the goals of this scoping review, critical appraisal tools were not used.

To synthesize results, we grouped articles into those that used quantitative, qualitative, and mixed methods. To gain insight into research gaps, we summarized measurement characteristics (e.g., whether the study used a previously-established or newly-created measure; whether the assessment referenced scanxiety or scans specifically or instead included general measures of anxiety/stress) and the timing of assessments with respect to the scan. In addition, we grouped articles according to period in the cancer continuum (during vs. post-treatment) and summarized the cancer types and stages of the study samples’ participants. To gain insight on promising intervention strategies, we reviewed articles’ results for correlates of scanxiety to identify modifiable characteristics, and applied expertise in clinical psychology to suggest how these patterns could inform intervention development.

## 3. Results

**Figure 1:**
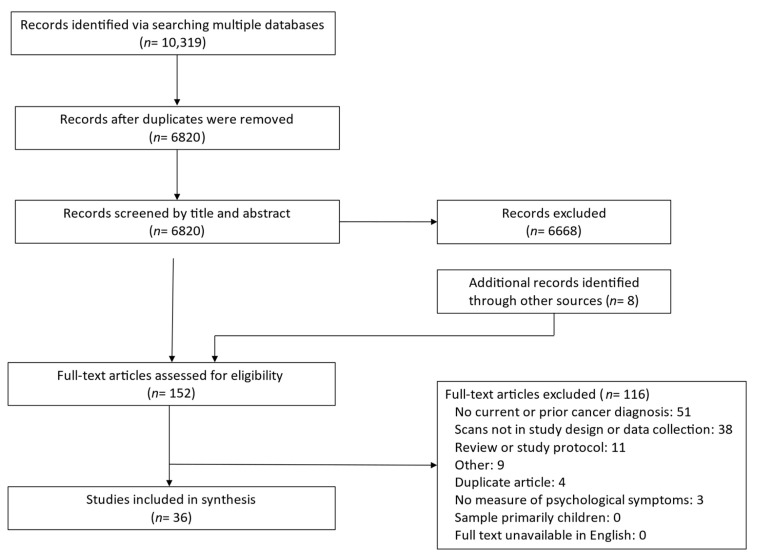
PRISMA diagram.

The systematic search of multiple databases returned 10,319 references, as summarized by the PRISMA flow diagram in Figure 1. After removing duplicates, 6,820 titles and abstracts were screened. Of these, 152 full-text articles were evaluated for inclusion, including eight identified in the updated search. At the full-text review stage, articles were most frequently excluded due to a focus on individuals without current or prior cancer (such as those undergoing initial cancer screening) or due to not incorporating scans into the study design, data collection, or results. Ultimately, 36 full-text articles were included in the review.

### 3.1. Study Types and Designs

There were 22 quantitative articles, summarizing 8 cross-sectional [3,13,14,15,16,17,18,19,20], 12 longitudinal [13,14,15,16,21,22,23,24,25,26,27,28], and 2 intervention trial study designs [29,30]. There were 9 qualitative [2,25,26,27,31,32,33,34,35] and 5 mixed methods articles [21,22,28,36,37].

### 3.2. Definitions and Elements of “Scanxiety”

In five articles, authors explicitly defined scanxiety (Table 2; [2,3,17,29,31]). Several articles credited Bruce Feiler for first using and describing the term in his 2011 Time magazine article, who wrote that “scans are like revolving doors, emotional roulette wheels …” and “[scans] engender ‘scanxiety’ as they approach” [1]. A common component of these definitions included a time course, suggesting that distress or anxiety can occur before, during, and after imaging procedures. One article explicitly emphasized that scanxiety is “normal” [29]. Among articles that did not define scanxiety, several did include a definition of a closely-related concept (e.g., fear of recurrence), or referenced that aspects of the scan experience can be stressful (e.g., “anxieties can be heightened around the time of a scan…” [18]).

**Figure 2:**
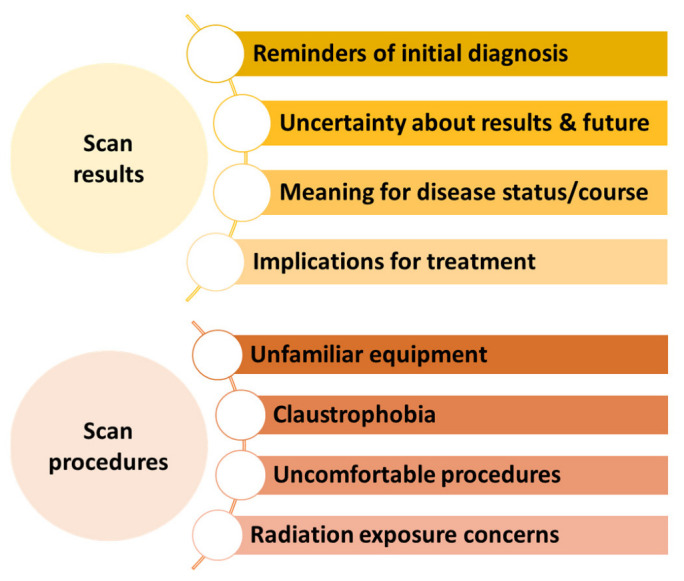
Dimensions of scanxiety, clustering around scan procedures and scan results.

Several articles’ narratives described specific elements of the scan experience that may be anxiety-provoking for patients. Most commonly, articles noted the uncertainty and fear associated with what the results would show, and the implications of scan results for their disease status and treatment [13,17,20,21]. Authors also described anxiety-provoking aspects of the imaging procedures, such as specialized or unfamiliar technology/equipment [13,21], discomfort [21], and claustrophobia arising from being in an enclosed space [13,21]. Concerns about exposure to radiation from the procedures were also noted [13,38]. Given the variety of concerns described, there are likely individual differences in what part of the scan experience is considered most stressful. These components suggest that scanxiety is multi-faceted, and people with cancer may have multiple and differing concerns relating to scans (Figure 2).

Quantitative results in several articles provided further detail on aspects of scanxiety. In a quantitative study of patients undergoing low dose PET/CT with 18-F-fluor-2-deoxi-D-glucose (^18^F-FDG), patients rated pre-selected reasons for their scanxiety [13]. Before a scan, 79% rated that they were most anxious about the results; others reported that they were most anxious about the scan procedure (12%), their illness (3%), or other areas (6%). This pattern did not change much afterwards in the post-scan assessment, with 87% reporting being most anxious about the scan results [13]. Other articles focused on the scan procedures (per the specific study goals), but did not appear to ask explicitly about anxiety related to the scan results. These articles illustrate additional concerns that patients may have around scans. For example, researchers focused on which experiences during MRI and PET/CT procedures were most stressful to esophageal cancer patients [39]. Following a series of scans, participants reported that body position in the scanner (52%) and waiting time before scanning (19%) were most stressful during PET/CT. The noise of the scanner (26%), scan time (22%) and body position (22%) were noted as stressful during the MRI. Comparing the ratings for MRI versus PET/CT procedures showed that anxiety did not differ between them [39]. In another quantitative study of cancer survivors in the United States who participated in the Health Information National Trends Survey (HINTS), 73% of respondents reported at least some worry about the effects of radiation from surveillance scans (i.e., medical imaging radiation; MIR), and 16% reported “a lot” of worry [38].

Qualitative findings also indicated a range of components of the scan experience that were stressful. For example, in focus groups, post-treatment breast cancer survivors described that undergoing scans reminded them of their initial diagnosis and promoted fears of recurrence [32,40]. Similarly, individual interviews with survivors of several cancer types suggested that scans served as a reminder of the “fragility of survivor status” and resulted in “iatrogenic uncertainty” [33]. Claustrophobia [41], exposure to radiation [18], pain and discomfort associated with the procedures [18,32], and receiving information from electronic reports [31] were also mentioned as sources of anxiety in qualitative interviews. Among people living with advanced cancer, results from open-ended questions and semi-structured interviews suggested that “most anxiety centered around the scan result and its implications” [29] and that the procedures themselves were a less significant driver of anxiety [2]. Taken together, these findings suggest that multiple aspects of scans may be anxiety-provoking for patients (Figure 2).

### 3.3. Populations Examined

Below, we summarize descriptive statistics of cancer-related characteristics from the articles’ samples. The number of articles summarized for these characteristics often exceeds the total number of included articles (n = 36) because some included individuals across multiple cancer types, stages, or other characteristics.

In 23 articles, the sample comprised individuals with a single cancer type [3,14,16,18,23,24,25,26,27,28,30,32,34,36,37,39,40,41,42,43,44,45,46]. In 13 articles, individuals with multiple cancer types were included in the sample [2,13,15,17,20,21,22,29,31,33,38,47,48]. In two articles, individuals’ cancer type was not specified [20,21]. Across articles, samples most commonly included people with breast (n = 18; [8,13,14,24,25,26,27,28,29,32,33,37,38,40,42,44,45,48]), lung (n = 10; [2,3,13,15,17,29,33,34,41,48]), or gastrointestinal (n = 9; [2,15,29,31,33,36,39,46,48]) cancer. Most articles included people with solid tumors, while fewer (n = 5; [13,18,22,33,48]) included those with hematological malignancies (lymphoma or leukemia).

Seventeen articles included individuals living with cancer (i.e., those with current cancer, typically in active treatment [2,3,15,16,17,23,29,30,31,34,37,38,39,41,46,47,48]), and 19 included individuals in the post-treatment period [14,18,22,24,25,26,27,28,30,31,32,33,36,38,40,42,43,44,45]. Three did not specify participants’ status along the cancer continuum [13,20,21]. People with non-metastatic cancer were included in 22 articles [2,3,14,16,23,24,25,26,27,28,29,30,31,32,36,40,41,42,43,44,46,47], and those with metastatic cancer were included in 11 articles [2,3,15,17,29,31,34,37,42,46,47]. Specifically, 9 [14,24,25,27,28,30,32,40,42], 14 [14,16,24,25,26,27,28,32,36,40,41,42,43,46], 12 [14,24,25,26,27,28,32,36,40,41,42,46], 16 [2,3,14,24,25,26,27,28,29,32,36,40,41,42,46,47], and 11 [2,3,15,17,29,31,34,37,42,46,47] articles included people with Stage 0, I, II, III, and IV disease, respectively. Thirteen articles did not specify the stage of participants’ cancer [13,18,20,21,22,23,31,33,38,39,44,45,48].

The reasons that participants were undergoing scans varied. Often, people who were undergoing scans for different reasons were included in the same studies. The most commonly listed purposes of scans were routine post-treatment monitoring to detect disease recurrence (n = 21; [13,14,18,21,22,24,25,26,27,28,30,31,32,33,36,40,42,43,44,45,48]) and routine monitoring to detect disease progression or treatment response (n = 15; [2,3,13,15,16,17,21,23,29,30,31,34,37,47,48]). Other articles included participants undergoing scans for post-diagnosis staging or treatment planning (n = 6; [13,21,39,41,46,48]. Only one article included individuals who were undergoing investigative scans for new symptoms [29]. The scans’ purpose was not specified in two articles [20,38]. Articles included a wide variety of scan types (Table 3).

### 3.4. Measurement Methods

Measurement of psychological symptoms included diverse concepts, such as distress, anxiety, stress, fear of cancer recurrence, and emotional well-being. Some articles included assessments that were specifically worded with reference to a scan, while others included a measure of general symptoms without a specific focus. For the purpose of consistency and brevity, and to reflect that these concepts were measured around or associated with scan procedures, we refer to all of these psychological symptoms as “scanxiety” in this paper.

Quantitative measures were used in 27 articles, including the five mixed methods articles [3,13,14,15,16,17,18,20,21,22,23,24,25,26,27,28,29,30,37,38,39,41,42,43,46,47,48]. Of these, 10 articles used more than one quantitative measure [14,15,20,25,27,29,30,41,42,48]. The most frequently used quantitative assessment tools were single-item measures that were developed for the purpose of the given study. Such measures were used in eight articles [21,25,29,39,42,46,47,48]. Across articles, there were 28 unique assessment tools; see Table 4. There were 14 scan-specific measures (used across 17 articles) that were worded specifically with respect to scans, and 14 general measures (used across 24 studies) that assessed psychological symptoms without a specific reference to scans.

Among the articles that used quantitative measures, 20 provided information about the timing of survey administration with respect to the scan or scan results discussion [3,13,14,15,17,20,21,23,24,25,26,27,28,29,30,39,41,46,47,48]. In seven articles, the timing of survey administration was unspecified or was not timed with respect to the scan [16,18,22,37,38,42,43]. In four articles, scanxiety was assessed only before a scan procedure or scan results discussion [3,20,26,47]. In five articles, scanxiety was assessed only after a scan or scan results discussion [17,29,30,39,46]. In 11 articles, scanxiety was assessed both before and after a scan procedure or scan results discussion [13,14,15,21,23,24,25,27,28,41,48].

Fourteen articles used qualitative methods, including the five mixed methods articles that also incorporated quantitative measures above [2,18,29,30,31,32,33,34,36,37,40,41,44,45]. Of these, 13 articles used semi-structured interviews (n = 4 in focus groups [30,32,36,40], n = 9 individually [2,18,31,33,34,37,41,44,45]) and one used open text box responses [29]. In nine articles, the interview topic guides or open text questions explicitly asked about scanxiety or emotions with respect to scans [2,18,29,30,31,32,34,40,41]. In the other five articles using qualitative methods, responses and themes regarding scanxiety emerged when asking other questions about cancer survivorship, follow-up care, and quality of life, and were thus reported in the results [33,36,37,44,45].

### 3.5. Consequences of Scanxiety

Of the articles reviewed, eight quantitatively investigated [3,17,22,24,26,29,41,47] and five qualitatively summarized [2,18,33,44,45] the effects of scanxiety. These articles highlighted effects on quality of life, somatic symptoms, receipt of follow-up care, and other healthcare experiences.

#### 3.5.1. Quality of Life

Several articles highlighted links between scanxiety and poorer quality of life. Patients with metastatic cancer who reported scanxiety had poorer quality of life on the EORTC-QLQ-30 than those who did not [17]. In another article, advanced lung cancer patients who experienced higher scanxiety reported poorer quality of life on the FACT-L than those with lower scanxiety [3]. By examining the subscales of the FACT-L, the authors concluded that this relationship was primarily driven by an association between scanxiety and emotional well-being. Consistent with these findings, metastatic cancer patients’ anxiety as assessed before a scan results discussion was associated with poorer psychological well-being on the McGill quality of life questionnaire [47].

#### 3.5.2. Somatic Symptoms

Findings from included articles suggested that scanxiety is linked to somatic symptoms. For example, advanced cancer patients reported that they experienced trouble sleeping (32%), feelings of dread (29%), poor concentration (26%), irritability (25%), and restlessness/agitation or tension (24%) due to scanxiety [29]. Other somatic symptoms including pain, low appetite, and racing heart, among others [29]. In a study of women undergoing mammograms following breast cancer treatment, participants with higher fear of cancer recurrence experienced poorer sleep both immediately before and one week after their mammograms compared to those with lower fear of cancer recurrence [24]. Similarly, advanced cancer patients described difficulty sleeping, fatigue, irritability, poorer concentration, and lower motivation for daily activities in qualitative interviews [2].

#### 3.5.3. Receipt and Experiences of Follow-Up Care

There were complex relationships between scanxiety and receipt of follow-up care, and the direction of the effects differed by article. For example, in a qualitative study of post-treatment lymphoma survivors, some reported that fear of recurrence motivated them to complete follow-up care, while others said it was a barrier [18]. These different patterns were also observed in quantitative studies. Among people with metastatic cancer, 16% reported that they had delayed follow-up care due to scanxiety [17]. A similar pattern was observed in post-treatment breast cancer survivors, such that those with higher levels of anticipatory anxiety were less likely to undergo mammography in the following year than those with lower levels of anticipatory anxiety [26]. Specifically, compared to women without anticipatory anxiety, those with median levels of anticipatory anxiety were 32% less likely to undergo a mammogram in the next year. Women with higher anticipatory anxiety also reported more negative responses to pain during the mammogram, which partially explained the relationship between anxiety and mammogram adherence in a mediation model. Findings from a latent class analysis of adult survivors of childhood cancers indicated a different pattern [22]. Those in the “worried” latent class were more likely to report completing mammography, as well as other aspects of follow-up care including ECG and bone densitometry, within the recommended time frame than those in other latent classes. Finally, in a study involving a series of three scans, baseline anxiety scores did not differ between those who went on to complete all of their scans in the series versus those who did not [41].

Qualitative findings added further context to these relationships. Following breast cancer treatment, some women noted that concern about recurrence was a motivator for completing follow-up surveillance care, while others reported that fear of recurrence was a barrier to follow-up care [45]. Another article highlighted that mammography was perceived as reassuring, and thus “worth the discomfort it may cause”, suggesting that anxiety was not perceived as a barrier to completing follow-up mammograms [44]. In survivors with various cancer diagnoses and cancer statuses, it was noted that follow-up tests (e.g., mammograms) were reassuring for some, while at least one participant noted that they did not experience this relief and considered discontinuing follow-up [33].

The effects of scanxiety on other healthcare experiences were examined in two articles. Among advanced cancer patients, those with higher anxiety prior to a scan results discussion were less likely to report their recently-discussed scan results accurately (compared to their oncologists’ reports) than those with lower anxiety [47]. In lung cancer patients, anxiety was not related to motion artifacts during the scan, which can affect the quality of the imaging for interpretation [41].

### 3.6. Correlates of Scanxiety

Of the articles reviewed, 21 quantitatively evaluated factors that were associated with scanxiety [3,13,14,15,16,17,18,20,21,23,24,25,27,28,29,31,38,39,41,46,48], and 12 described qualitative themes regarding such factors [2,18,29,30,31,32,34,37,40,41,44,45]. Findings are synthesized below, grouped by factors that reflect sociodemographic characteristics, cancer-related characteristics, scan-related factors, timing of assessment, clinic- and system-related factors, and psychological and behavioral factors.

#### 3.6.1. Sociodemographic Characteristics

In two articles, women reported higher scanxiety or were more likely to endorse scanxiety compared to men [20,29]. In Abreu and colleagues’ study, there were no gender differences in scanxiety prior to the scan procedure, but men reported higher scanxiety than women afterward [21]. Gender was not associated with scanxiety in four articles [3,13,18,38], although there was a trend toward higher scanxiety in women in two of these articles [3,18].

Age was not associated with scanxiety in most articles [3,13,15,23,38]. In a study of advanced cancer patients, younger participants were more likely to endorse experiencing scanxiety than older participants [29]. Similarly, in people undergoing active surveillance or primary intervention for kidney cancer, those who were younger had poorer mental health scores than those who were older [16].

Few articles examined the relationship between scanxiety and race or ethnicity. In advanced lung cancer patients, there was no significant relationship between race or ethnicity and scanxiety [3]. Among cancer survivors in the HINTS study, Black individuals and those who were foreign-born were more worried about exposure to medical imaging radiation than white and US-born individuals, respectively, in unadjusted analyses, but these relationships did not remain in adjusted models [38].

While education level was not associated with anxiety in three articles [3,13,21], several articles suggested that scanxiety may be worse among those with lower education levels. In the HINTS study, cancer survivors with lower educational attainment reported more worry about medical imaging radiation than those with higher educational attainment in unadjusted analyses, but this relationship was weakened in adjusted models [38]. Similarly, people with lower education had higher scanxiety when undergoing imaging than those with higher education [20]. In a study of advanced cancer patients, those with lower education levels were more likely to endorse scanxiety than those with higher education levels, but interestingly, health literacy was not associated with scanxiety [29]. In this study, participants who lived in more remote locations were also more likely to report experiencing scanxiety than those who lived in more urban locations [29].

Other sociodemographic factors were not associated with anxiety or stress around the time of scans, including smoking status [3], income [3], and relationship/marital status [3,29]. Medical imaging radiation worry was not associated with health insurance status, but those with lower income levels were more likely to report medical imaging radiation worry than those with higher income levels [38].

#### 3.6.2. Cancer-Related Characteristics

Several articles included individuals with and without cancer and examined whether scanxiety varied with cancer history. Using daily data collection before and after mammograms, Porter and colleagues observed that breast cancer survivors had a slightly greater increase in daily stress from baseline to mammogram day compared to control women without a cancer history [25]. On the other hand, patients undergoing imaging tests for cancer had lower anxiety levels on the day of the exam than those who underwent imaging for other health conditions [20]. In a three-week daily diary study, participants with cancer reported significantly higher fear of cancer recurrence across the study period and greater peak levels of fear of cancer recurrence on the day of their mammogram compared to their spouses [27]. Another study indicated a different pattern, in which 71% of patients and 81% of their caregivers reported scanxiety symptoms on a Greek version of the IES-R, though the score cutoff and statistical significance of this comparison was not reported in the conference abstract [17].

In two quantitative articles, time since diagnosis was not related to levels of scanxiety [3,15]. Other articles suggested a complex relationship. Among advanced cancer patients, those who were diagnosed less than a year ago endorsed similar rates of experiencing scanxiety (vs. not experiencing scanxiety) compared to those who were diagnosed more than a year ago [29]. However, in the subset of participants who endorsed scanxiety, those who were diagnosed less than a year ago rated their peak anxiety as higher than those with a longer time since diagnosis [29]. In a cross-sectional study, patients with a recent diagnosis within six months or less had higher levels of scanxiety than those who had been living longer with cancer [17]. However, in the same study, participants also reported that scanxiety did not dissipate over time [17]. In the HINTS study, those who had completed treatment within the past 10 years reported higher worry about medical imaging radiation than those who had completed their treatment more than 10 years ago [38]. Qualitative findings similarly highlighted that scanxiety may decrease over time for some people with cancer, but not for others. For example, some patients with metastatic lung cancer reported that their anxiety diminished as their condition stabilized, while others reported that scanxiety is “always there” [34]. Among veterans with bladder cancer, some noted that anxiety lessened over time with repeated procedures, and with meeting “experienced” patients who had been living with cancer for a longer period of time [30].

Few articles investigated differences in scanxiety based on cancer stage, cancer type, treatment type, or health status. Advanced lung cancer patients’ scanxiety did not vary according to their cancer histology, stage, or the presence of an actionable tumor mutation [3]. Patients with progressive lung cancer did not experience significantly higher levels of scanxiety than those whose disease was stable or improving [3]. On the other hand, cancer survivors with poorer self-rated health had higher levels of medical imaging radiation worry than those who rated their health better [38]. Larger proportions of those with breast and lung cancer reported higher medical imaging radiation worry compared to other cancer types, and those who received radiation treatment were more likely to report worry than those who did not [38]. Among those with advanced cancer, those with breast cancer were more likely to report experiencing scanxiety around a recent scan compared to those with other cancers [29]. Clinical trial participation was not associated with scanxiety [15].

#### 3.6.3. Scan-Related Characteristics

The number of prior scans or frequency of scans was not reported in most articles we reviewed. In two articles, anxiety level was not significantly different for those who were undergoing the ^18^F-FDG PET/CT scan procedure for the first time compared to those who had completed it previously [13,21]. Similarly, among patients who were undergoing a series of multiple scan types, including MRI and CT, anxiety was not statistically different between the first and second MRIs [41]. Nevertheless, in qualitative interviews with advanced cancer patients, increasing familiarity with the procedures was noted to be linked to lower scanxiety [2].

Findings were inconsistent for whether scanxiety varied by scan type. For example, among lung cancer patients undergoing CT and MRI scans, state anxiety scores did not differ significantly by scan type [41]. However, in the semi-structured interview component of this mixed methods study, participants described that MRI scans prompted more anxiety, claustrophobia, and discomfort than CT scans [41]. In Goense and colleagues’ study, participants underwent both MRI and PET/CT scans; they concluded that anxiety did not vary significantly by scan type [39]. Follow-up regimen type was also not associated with differences in mental well-being between kidney cancer patients who received active surveillance including regular imaging (CT, MRI, and ultrasound) and those who received primary intervention [16]. On the other hand, three studies found group differences in anxiety according to scan type, with anxiety being higher for MRI (vs. CT) [20], for endoscopic ultrasonography (vs. cervical ultrasonography, CT, or PET) [46], and for rigid (vs. flexible) cystoscopy [23].

Several articles examined characteristics of the scan experience. For example, one study indicated that patients’ experiences during the scan (e.g., discomfort, difficulty) were associated with higher anxiety directly following the procedure [13]. Similarly, focus groups with breast cancer survivors suggested that pain and discomfort during mammogram procedures were a source of anxiety [32]. The reason that the scan was being conducted was not related to anxiety [13,21]. Interestingly, a study of advanced cancer patients did not indicate a significant association between type of result received (stable or better vs. progressive) and retrospective anxiety reports [29]. Patients’ expectations about the nature of the results were also not associated with pre-scan distress levels [15]. Among advanced lung cancer patients attending an appointment, those who reviewed a recent scan during the visit did not have significantly higher scanxiety severity compared to those who did not review a scan at the visit [3].

#### 3.6.4. Timing of Assessment

Articles with longitudinal designs examined the effect of time by comparing scanxiety at different points in the scan experience. In two articles, anxiety increased from baseline to pre-mammogram [14,27]. In six articles, anxiety decreased from pre- to post-scan procedure [14,21,23,24,27,41]. In one article, participants experienced higher anxiety following the scan compared with their pre-scan anxiety [13]. In at least two articles [14,24], participants had received their results by the post-scan time point. However, the timing of results was not always clear with respect to the study assessments.

#### 3.6.5. Other Clinic or System-Level Factors

In qualitative articles, the waiting period between the scan procedure and receiving scan results was often described as the most distressing period [2,30]. Participants described that anxiety was present until the results of the scan were known [40,44]. In separate articles of post-treatment breast cancer and lymphoma survivors, both research teams noted that the days between the scan and receiving results were particularly stressful and reminded participants of the time period of when they were first evaluated for and diagnosed with cancer [40,45]. Wait times may be shortened when receiving results electronically (e.g., via patient portals), which was perceived positively by some patients [37]. Qualitative findings also suggested that other stressors arose with this option for some patients, such as the potential to feel worse after a “bad result” or to need reassurance from an oncologist [31,37].

Although this waiting period was commonly noted as anxiety-provoking in qualitative articles, few quantitative articles examined this factor. Advanced cancer patients who waited more than two days for their scan results were more likely to retrospectively report experiencing scanxiety [29]. With respect to results delivery, advanced cancer patients who were notified about their results by their preferred provider had greater decreases in their distress following this discussion than those who received results from a provider that differed from their preference [15]. However, participants’ distress before imaging and after results was not associated with whether their preferences were met for the time frame or method for delivering results [15]. Among lymphoma patients, those who reported a worse patient–doctor relationship had higher anxiety than those who reported a better relationship, although this assessment was not timed around scans specifically [18]. On the other hand, worry about medical imaging radiation was not associated with physician trust in a heterogeneous sample of cancer survivors [38].

In another article, advanced cancer patients rated clinic-level factors as helpful around the time of the scan or scan results [29], although the statistical relationships between these factors and anxiety were not tested specifically. Several factors reported to be helpful around the time of scans were having experienced (81%) and friendly (88%) staff conducting the scan procedures, knowing what to expect about the procedures (82%), and undergoing the scan at a familiar location (i.e., close to home or at the center they received treatment, 71%). Around the time of receiving scan results, several factors reported to be helpful were the availability of scan results at the time of their oncologist appointment (91%), receipt of results from their oncologist in clinic (90%), and discussing the treatment plan in the same visit (81%) [29].

#### 3.6.6. Psychological and Behavioral Characteristics

Some articles indicated that scanxiety was more common among those with certain baseline psychological factors. Those with higher general anxiety symptoms and fear of cancer progression were more likely to endorse experiencing scanxiety [29]. General anxiety symptoms were also associated with patients’ ratings of peak anxiety around the time of the scan [29] and distress on the day of imaging [15]. In a study of people with bladder cancer, those with higher baseline anxiety also had higher anxiety following the cystoscopy procedure [23]. On the other hand, this relationship was not observed between elevated general anxiety and worry about medical imaging radiation [38]. The likelihood of reporting scanxiety in the context of a recent scan did not differ for those with and without clinical depression [29]. In a daily diary study, patients with higher threat sensitivity (i.e., a tendency to have stronger responses to threatening cues or situations) had higher fear of cancer recurrence on the day of their mammogram and slower rates of recovery (decreases in fear of cancer recurrence) following the mammogram than those with lower threat sensitivity [27].

Other articles examined how health behaviors and coping strategies were related to scanxiety. For example, people who searched for information prior to their scan did not have higher levels of anxiety compared to those who did not [21]. In bladder cancer patients, low perceived control over the procedures was noted, and the ability to watch the procedure in real time was noted to help improve feelings of control [30]. Empowerment or self-efficacy in managing their health by undergoing the procedure was noted as a positive factor [30]. However, in a trial testing a self-management approach following treatment for endometrial cancer, women randomized to patient-initiated follow-up (self-referral if alarming symptoms occurred) had smaller decreases in fear of recurrence compared to control participants receiving regularly-scheduled hospital-based follow-up [43]. Articles also summarized ways of coping; patients reported using a wide range of strategies, including distraction, relaxation, positive self-talk, and self-management of care [2,41] to tolerate scans and manage scanxiety. Among post-treatment breast cancer survivors, greater confidence in one’s ability to cope with potential recurrence was associated with lower fear of recurrence around surveillance [14]. Following a PET-CT scan, state anxiety scores were lower among cancer patients who were assigned to listen to music immediately before a PET-CT scan compared to those assigned to a control condition [48].

Several articles examined how social relationships were associated with scanxiety. Although emotional and instrumental social support were not associated with pre-scan distress, patients who reported greater social isolation had higher distress on the day of their imaging compared to their socially connected counterparts [15]. In a dyadic design using daily diary methods, partner responsiveness (i.e., the degree to which one perceives their partner to respond with genuine interest and care) and capitalization attempts (i.e., the process of sharing good news with one’s partner) were not related to fear of cancer recurrence before a mammogram [28]. Contrary to hypotheses, patients whose partners were more responsive had higher fear of cancer recurrence on the day of the mammogram than those with whose partners were less responsive [28].

## 4. Discussion

In this scoping review, we identified and synthesized findings from 36 articles on scanxiety among adults diagnosed with current or prior cancer. The articles included a notable breadth of individuals across cancer types, disease status, and stage, suggesting that scanxiety occurs across the cancer continuum. Our synthesis indicated that there are various components of the scan experience that prompt anxiety, which may differ across individuals. These components appear to cluster around aspects of the scan procedure itself and around the uncertainty associated with high-stakes results. The waiting period between the scan procedure and receipt of the results was described as particularly stressful, although few quantitative articles captured this period of time or described how results were delivered. Our review also summarized the measures and methods used in scanxiety research. The assessment tools used to measure scanxiety varied widely, with 28 unique assessment tools, and often relied on newly-created single items for study-specific purposes. While this leads to a rich literature to examine scanxiety from different angles, this variation limits conclusions about levels or consistent factors relating to scanxiety. Strengths of our approach include the large literature base screened and reviewed, the inclusion of both quantitative and qualitative research designs, and the focus on cancer survivorship. Although comprehensive searches were conducted in July 2020 and March 2021, an adjusted search approach limited to Pubmed was conducted for the final period of this review (March 2021 to July 2022), a limitation. Below, we discuss how the findings of this review could be used to inform future research directions and to generate hypotheses and approaches for interventions (Table 5).

### 4.1. Current Research Findings, Methods, and Gaps

Conceptual clarity varied across articles. Few articles provided a specific definition of scanxiety. This may have been because research questions on scanxiety were often secondary to other study aims. With limited conceptual clarity, it is not surprising that many different survey measures were used, with little harmonization across articles. To advance the science of scanxiety, there is a need to examine how it relates to closely-related constructs that may be exacerbated around scans, such as fear of recurrence or progression, anticipatory anxiety, and state anxiety. Determining if these constructs are largely the same, strongly correlated, or composed of different components would help to harmonize measurement strategies for indexing anxiety around scans. Because precise measurement methods are especially needed for assessing the impact of interventions, a strong interim approach might be to utilize measures that are worded specifically in reference to a scan (Table 2), such as the Impact of Events Scale (six items) and the modified Distress Thermometer.

Relatedly, neighboring literature on anxiety while awaiting other types of tests (e.g., blood tests) or routine follow-up examinations should be evaluated to determine if experiences are similar during these waiting periods. For example, many patients receive key prognostic information about their disease from blood tests such as carginoembryonic antigen (CEA), prostate-specific antigen (PSA), or cancer antigen 125 (CA125) levels. As technology becomes more sophisticated, additional blood tests (e.g., cell-free DNA, or “liquid biopsy” for mutational analysis of tumors) bring with them the potential for additional distress to patients. The extent to which similar processes of heightened anxiety and uncertainty occur around these types of tests is needed. Based on the definitions and elements of scanxiety from our review, we suggest that this experience may not be limited to scans, but instead may occur in the context of other types of follow-up tests that involve uncomfortable procedures, waiting for results, and uncertainty around implications for disease status. During the article screening process, we identified articles that focused on different types of tests or follow-up without mention of imaging, but we did not review these articles based on our research objectives and inclusion criteria. Because the search terms were designed to focus on imaging-related articles, our review of literature on other testing methods would not have been comprehensive or representative. However, we identified additional articles that could be relevant to the concepts raised here, an area for future work. Findings might also inform considerations for administering existing measures or developing new ones. For example, measures or terminology that apply to anticipatory anxiety around multiple types of tests may be needed. Scanxiety is currently used to describe these processes specifically around scans, and we are not aware of broader terms that encompass anxiety around new or alternative tests that result in similar prognostic information.

The characteristics of the samples and designs in our review also point to areas in which research can be extended. For example, most of the current scanxiety literature focuses on those with solid tumors, and fewer articles included individuals with hematological malignancies. In addition, articles typically focused on individuals who were undergoing routine, scheduled scans to detect progression, treatment response, or recurrence. Only one study included individuals who were undergoing investigative scans due to new symptoms, but it seems that anxiety would likely be even higher in this context. Third, scanxiety among family members/care partners has been under-studied. Research is needed to determine their anxiety levels and needs for psychosocial support, and interactions between these factors and the patient’s experience around the time of scans. Broader literature suggests that family members’/care partners’ general anxiety is significant and can be higher than patients’ anxiety [49,50], so it is possible that periods around scans are even more stressful for them. Another future direction is to determine how delivery of scan results may impact scanxiety, especially in the context of newer formats such as direct release through patient portals [31,51]. Given that a common focus of scanxiety was on what the scan results showed, additional research that examines how results are delivered and what they showed would be useful. These details about results delivery were rarely included in the articles reviewed, with several exceptions [3,14,15,29,47]. Finally, research addressing how scanxiety affects day-to-day life, clinical experiences, and follow-up care receipt is limited. Because existing articles highlighted that scanxiety drives some patients to seek greater follow-up care and others to seek less, there is a need for research to test “for whom” and “how” scanxiety affects follow-up care. Both patterns can be problematic but likely require different management strategies.

Our review also highlights areas for methodological improvements in scanxiety research. For example, psychometrics of quantitative measures were rarely provided. Overall, the description of timing of measurements with respect to the scan procedure and the results delivery could also be improved. Longitudinal assessments often included pre- and post-scan time points, but it was often unclear when scan results were delivered with respect to the post-scan assessment. To the extent possible in a given study design, it would be useful to report the nature of the test results as well as whether, when, and how results were delivered. Prospective designs that captured the period between the scan procedure and receipt of the results were especially limited. Future research on this time period is needed, given that it was identified as the most stressful by patients in qualitative articles. In addition, methods that allow for repeated measures around these times, such as daily diary designs or ecological momentary assessment, could help to elucidate fluctuating patterns of anxiety over time with respect to these events. While these methods are acceptable and feasible in young survivors of childhood cancer around the time of scans [52], evaluating their acceptability and feasibility during these time periods in older survivors and those with active disease would be useful.

Based on our experience with the screening phase of this project, description of the components of clinical follow-up procedures could be improved in future articles. Some of the screened abstracts and articles had a focus on routine follow-up, but did not describe what procedures were included in these visits, or whether they involved scans or other types of tests. We excluded these articles if scans were not mentioned. However, operationalizing the types of tests or procedures in regular follow-up could potentially expand the pool of scanxiety articles available to draw upon. Accordingly, it is possible that the group of available articles is actually larger than what was reviewed here. In addition, in the broader cancer literature on anxiety and stress, it is possible that symptoms are assessed at the time of tests or follow-up visits while awaiting results, and thus affected by scanxiety. Future work on anxiety could be advanced by including questions about whether participants are awaiting upcoming tests, scans, or follow-up care to help interpret fluctuations in anxiety.

### 4.2. Promising Approaches to Intervention

To develop and test interventions for scanxiety, a key step is to learn which individuals are most at risk for scanxiety. While more research is needed and findings were not always consistent, our review indicated that women and those with lower education levels, shorter time since diagnosis, and greater levels of baseline anxiety may be more likely to experience scanxiety, while associations with other factors were less consistent. If these patterns hold true in future research, this information could be used to emphasize the inclusion of these individuals in the intervention development process, tailor strategies to their needs, and prioritize them for intervention delivery. For example, screening for baseline characteristics (e.g., general anxiety or fear of recurrence) could be used to inform which patients may be likely to need or benefit from additional support around the time of scans. Future research supporting these patterns could also inform intervention components. For example, it is possible that helping patients manage high levels of general anxiety could have a positive impact on their scan-specific anxiety. However, we did not analyze these patterns using meta-analysis, and the measures and time points examined were quite heterogeneous. Given these limitations, our synthesis can be used to generate hypotheses rather than to draw firm conclusions on whether these factors are related to higher scanxiety. Further research is needed to determine which patients may be most prone to increases in anxiety around the time of scans.

Few articles examined modifiable or theory-driven individual-level factors to inform intervention targets that mitigate scanxiety, another area for future work. Nevertheless, the existing literature can be used to identify promising intervention approaches. For example, a study of post-treatment breast cancer survivors investigated whether factors from the cognitive-behavioral model were related to anxiety. Women who were more confident in their ability to cope with potential recurrence experienced lower fear of cancer recurrence around their surveillance mammograms than those with lower coping self-efficacy [14]. These findings suggest that interventions to strengthen peoples’ coping skills for how they might handle the potential “bad news” of recurrence might be useful, as opposed to reassuring oneself that risk is low (which was associated with greater anxiety in the study). Because the waiting period before receiving results is often uncontrollable and described as the most stressful period, strategies to pass the time during this difficult window might be especially useful. For example, advanced cancer patients described using strategies such as distraction and pleasant activities to cope with scanxiety [2]. Similarly, engaging in enjoyable flow activities that fully occupy one’s mind could make the waiting period more tolerable [53].

When developing and testing interventions, researchers should consider that anxiety may center around the scan procedures themselves, around the uncertainty associated with what the results may show, or both. It is likely that different types of intervention strategies may be needed to address these various components effectively. For example, a patient who is primarily concerned about the procedure due to claustrophobia may benefit from cognitive behavioral therapy, including exposure exercises. This approach would likely be ineffective and inappropriate for someone whose primary concern is the uncertainty of results. Short interventions around the time of scans, including real-time strategies employed in the waiting area or during scan time, may be beneficial and acceptable for those with procedural anxiety. Just-in-time micro-interventions represent a promising approach that may be well suited for these moments [54]. On the other hand, a common focus of scanxiety is the ongoing uncertainty associated with the possibility of recurrence or progression, or anxiety-provoking reminders of initial diagnosis. Interventions that are employed outside of the scan procedure itself, perhaps in the lead-up to the scan or emphasized during the waiting period between the scan and results receipt, may be better suited to address anxiety arising from uncertainty about the results. In this case, people may be expected to benefit from interventions that promote meaningful engagement in activities, mindful awareness, coping with fear of progression or recurrence, or acceptance-based strategies. As research on the most beneficial strategies to support those with scanxiety emerges, a helpful approach could be to offer several different evidence-based stress management tools [35], such that patients may practice those that resonate most with them.

This review also suggests that addressing clinic- and system-level factors could help mitigate scanxiety. For example, findings from qualitative articles repeatedly suggested that wait times, especially the waiting period between the scan procedure and receipt of the results, was particularly anxiety-provoking for patients. Shortening wait times to results could shorten periods of heightened anxiety for patients. Strategies that improve patients’ ability to know what to expect, such as implementing a set routine for results delivery or pre-scheduling discussion appointments, may also be useful. While the direct release of results via patient portals may provide a way to shorten wait times, data also indicates that some patients experience heightened anxiety or confusion around technical terms when viewing these results independently; thus, they may benefit from additional support, education, or informed decision-making to determine if this is the right approach for them [31,37,51]. Other patients may benefit from addressing aspects of the scan procedure itself. Improving the scan experience (e.g., by making adjustments that reduce pain or discomfort) could help to alleviate procedural anxiety. Finally, clinicians may benefit from considering which patients could struggle most with scanxiety. Shared decision-making with such patients (e.g., considering a longer interval to the next scan) as well as communicating about how to act on (e.g., anticipated treatment decisions based on results) and cope with (e.g., by voicing unending support for the patient no matter what the results, offering additional psychosocial supports as available) the potential results are other promising directions for future research and clinical practice.

## 5. Conclusions

Our review—which involved screening and reviewing a large literature base, included quantitative and qualitative research designs, and focused on cancer survivorship—provides insight on the nature of scanxiety, research practices and gaps, and promising strategies for interventions. In summary, scanxiety occurs across the cancer continuum. It may focus on procedural or uncertainty-related components of the scan experience. Future work to determine the ideal measurement strategies and timing of assessments would advance the current understanding of scanxiety and intervention approaches to help manage it. Efforts to harmonize measures, examine the waiting period between scans and scan results, and identify intervention targets are particularly needed. Emerging literature indicates that some individuals may be more prone to elevated anxiety around the time of scans, and suggests promising approaches at the individual and clinic/system levels to help manage scanxiety. Given that scan experiences are common and repeated across the cancer continuum, strategies to support patients around these times have the potential to improve quality of life and other outcomes for those living with and beyond cancer.

## Figures and Tables

**Table 1:** Eligibility criteria for full text screening.

*Inclusion*: Participants: Adults (ages 18 and older) with current or prior cancer diagnosis Concept: Undergoing imaging tests for detecting disease progression or recurrence Context: Completed a measure of psychological symptoms while awaiting scans or scan results or provided retrospective recollection of such symptoms. Types of studies: Observational studies, case studies, qualitative studies, conference abstracts, and intervention studies (single-arm and randomized controlled trials)
*Exclusion*: Study sample composed primarily of children (mean sample age under 18 years), due to likely differences in scan experiences and assessment methods People undergoing initial diagnostic cancer screening (i.e., no current or prior cancer diagnosis) Scans or scan result discussions not considered in study design, measurement, or results Review articles or study protocols Full text unavailable in English

**Table 2:** Definitions of scanxiety listed in included articles.

“Ten years ago, Feiler coined the term, ‘scanxiety’ to describe the emotional distress that patients experience immediately before or after medical imaging [31].” “Scanxiety represents a complex array of negative and stressful emotions linked with cancer scans, and the uncertainties and fears that may accompany them [17].” “Scanxiety” or scan-associated anxiety describes the distress before, during, or after a cancer-related scan and was a term first coined by a patient writing for the Time magazine in 2011. … There are no agreed criteria that define scanxiety. Unlike anxiety disorders, which are characterised by excessive fear and anxiety, scanxiety is often considered a normal reaction to a scan. Scanxiety is a transitory emotional state, which is consistent with the concept of state anxiety [19]. Though scanxiety may not be pathological in the same manner as an anxiety disorder, scanxiety may be a negative experience that impairs quality of life [29].” “Distress leading up to, during and after an imaging scan has been termed ‘scanxiety’ [2].” “‘Scanxiety’ refers to the often-debilitating anxiety patients with cancer experience in the period surrounding imaging studies for their cancer [3].”

**Table 3:** Summary of included articles.

	Sample Characteristics				Scan Characteristics		Scanxiety Measures		
Citation	N	Cancer Type	Cancer Stage	Cancer Status	Scan Type	Scan Purpose	Assessment Tool	Scan-Specific vs. General Measure	Design; Assessment Time Points
Quantitative Studies									
Abreu et al., 2017 [21]	232	Multiple	Not specified	Not specified	F-FDG PET/CT	Staging/early post-diagnosis Detect progression Detect recurrence	Likert item (anxiety, 10-point)	Scan-specific	Longitudinal: Immediately pre- and post-scan procedure
Bauml et al., 2016 [3]	103	NSCLC	III, IV	Living with cancer	Examples given: CT, MRI, PET	Detect progression	Impact of Events Scale-6 item version (IES-6)	Scan-specific	Cross-sectional: Waiting room prior to medical oncology appointment (scans discussed for 73% of patients)
Bjelic-Radisic et al., 2017 [42]	284	Breast	0–IV	Post-treatment	Multiple aspects of follow-up: Mammography, breast ultrasound, abdominal ultrasound, chest x-ray, bone scintigraphy	Detect recurrence	Breast cancer psychosocial assessment screening scale (BC-PASS)Likert items (distress about follow-up components, 1–4) Likert items (anxiety and stress before follow-up visits; 0–10 and 1–4)	General (BC-PASS) Scan-specific (Likert items)	Cross-sectional: Not specified with respect to scan
Cox et al., 2013 [22]	613; varied by analysis	Multiple; prior childhood cancers	Not specified	Post-treatment	Mammogram, ECG (ultrasound or multi-gated acquisition scan), bone density scan (DEXA or CT)	Detect recurrence Other: Detect late effects	Three summed items on health fears (future health, cancer recurrence, finding a problem at follow-up visits; 1–5)	General	Longitudinal: Not specified with respect to scan
Derry et al., 2019 [47]	94	Multiple; solid tumors	III, IV	Living with cancer	Not specified	Detect progression	Single item (anxiety about diagnosis, not at all to completely)	General	Cross-sectional: Prior to scan results discussion appointment
Goense et al., 2018 [39]	27	Esophageal	Not specified	Living with cancer	MRI and PET/CT to predict clinical response to neoadjuvant tx	Staging/early post-diagnosis information	Likert rating (anxiety, 1–5)	Scan-specific	Cross-sectional: Directly following the last of several MRI and PET/CT scans
Grilo et al., 2017 [13]	81	Multiple	Not specified	Not specified	^18^F-FDG PET/CT	Staging/early post-diagnosis Detect progression Detect recurrence	State Trait Anxiety Inventory (STAI)-state	General	Longitudinal: Before and after scan
Hay et al., 2018 [38]	452	Multiple	Not specified	Living with cancer Post-treatment	Not specified; item asked about radiation from medical imaging tests such as x-rays, mammograms, radioactive dye, etc.	Not specified	Single item from HINTS on Medical Imaging Radiation (MIR) worry (not at all to a lot)	Scan-specific	Cross-sectional: Not specified with respect to scan
Jeppesen et al., 2018 [43]	214	Endometrial	I	Post-treatment	Ultrasound; other imaging as needed	Detect recurrence	Fear of Cancer Recurrence Inventory (FCRI)	General	Intervention trial: Not specified with respect to scan
Koinis et al., 2022 [17]	218	Multiple	IV	Living with cancer	Not specified	Detect progression	Modified Greek version of Impact of Events Scale (IES)-Revised	Scan-specific	Cross-sectional: Within one week after scan
Krajewski et al., 2017 [23]	100	Bladder	Not specified; non-muscle- invasive	Living with cancer	Cystoscopy	Detect progression	Hospital Anxiety and Depression Scale (HADS)	General	Longitudinal: Directly before procedure and within 7–10 days after
Lo Re et al., 2016 [20]	260 (41% were oncology patients)	Multiple	Not specified	Not specified	MRI, breast imaging (mammogram, ultrasound, breast MRI), x-ray, CT, ultrasound	Not specified	STAI-state STAI-trait	General	Cross-sectional: Before scan procedure (presumably in waiting area)
Martinez-Lorca and Martinez-Lorca, 2022 [48]	108	Multiple	Not specified	Living with cancer	^18^F-FDG-PET-TAC	Early post-diagnosis/staging Detect progression Detect recurrence	STAI Likert scale (subjective anxiety, 0–10)	General	Intervention trial: Directly before and after scan
McGinty et al., 2014 [24]	136	Breast	0–IIIA	Post-treatment	Mammogram	Detect recurrence	Cancer Worry Scale (CWS)	General	Longitudinal: Immediately before and one week after mammogram (following negative results)
McGinty et al., 2016 [14]	161	Breast	0–IIIA	Post-treatment	Mammogram	Detect recurrence	Two visual analog scale items about fear of recurrence CWS	General	Longitudinal: Seven time points; baseline, one month before, one week before, immediately prior to mammogram, and immediately after, one week after, and one month after receiving results
Morreale et al., 2020 [15]	100	Lung Gastrointestinal	IV	Living with cancer	CT or MRI	Detect progression/assess treatment response	Distress Thermometer HADS	General	Longitudinal: On imaging day and one week after receiving results
Patel et al., 2016 [16]	539	Kidney	I	Living with cancer	Axial imaging (CT or MRI), ultrasound	Detect progression	SF-12 QOL Questionnaire Mental Component Score	General	Longitudinal: Not specified with respect to scan
Porter et al., 2003 [25]	55 (34 with breast cancer and 21 without cancer)	Breast	0–III	Post-treatment	Mammogram	Detect recurrence	Daily stress (single item, 0–10) Psychological consequences questionnaire (PCQ)	General (daily stress) Scan-specific (PCQ)	Longitudinal: Daily measures for three days about one month before, day of, and day after mammogram
Shelby et al., 2012 [26]	204	Breast	I-III	Post-treatment	Mammogram	Detect recurrence	Stanford acute stress reaction questionnaire	Scan-specific	Longitudinal: Immediately before mammogram; adherence over next year
Soriano et al., 2018 [28]	57 couples	Breast	0–III	Post-treatment	Mammogram	Detect recurrence	Daily measure, six items adapted from Insight and Severity subscales of FCRI	General	Longitudinal: Daily measures over three weeks; two weeks before and one week after mammogram
Soriano et al., 2019 [27]	57 couples	Breast	0–III	Post-treatment	Mammogram	Detect recurrence	Daily measure, six items adapted from Insight and Severity subscales of FCRI PROMIS Anxiety Short Form	General	Longitudinal: Baseline anxiety; Daily measures over three weeks; two weeks before and one week after mammogram (results typically given same-day)
Westerterp et al., 2008 [46]	82	Esophageal	I-IV	Living with cancer	CT, PET, cervical ultrasonography, endoscopy, ultrasonography	Staging/early treatment planning	Likert item (anxiety; 1–5)	Scan-specific	Cross-sectional: two weeks after scan procedures
Qualitative studies									
Allen, 2002 [44]	6	Breast	Not specified; non-metastatic	Post-treatment	Mammogram	Detect recurrence	Semi-structured individual interview	General	Cross-sectional: Not specified with respect to scans
Brandzel et al., 2017 [40]	41	Breast	0–III	Post-treatment	Mammogram and breast MRI	Detect recurrence	Semi-structured interview in focus groups	Scan-specific	Cross-sectional: Not specified with respect to scans
Bui et al., 2021 [2]	16	Multiple; solid tumors	III, IV	Living with cancer	CT	Detect progression	Semi-structured individual interview	Scan-specific	Cross-sectional: Within four months after scan
Lai-Kwon et al., 2021 [34]	20	NSCLC	IV	Living with cancer	CT; PET	Detect progression	Semi-structured individual interview	Scan-specific	Cross-sectional: Within three months after scans
Mannion et al., 2022 [31]	20	Multiple	Not specified; included those with Stage IV cancer (80%)	Living with cancer Post-treatment	Not specified	Detect progression Detect recurrence	Semi-structured individual interview	Scan-specific	Cross-sectional: Not specified with respect to scans
Pascal and Endacott 2010 [33]	15	Multiple	Not specified	Post-treatment	Variety including mammograms and other scans	Detect recurrence	In-depth individual interview	General	Longitudinal: Two interviews over six months, timing not specified with respect to scans
Sterba et al., 2015 [36]	22	Colorectal	I-III	Post-treatment	Not specified; regular follow up including CT and colonoscopy	Detect recurrence	Semi-structured interview in focus groups	General	Cross-sectional: Not specified with respect to scans
Thompson, H.S. et al., 2006 [45]	10	Breast	Not specified	Post-treatment	Variable as part of follow-up care; e.g., mammogram, x-ray, sonogram, CT, PET	Detect recurrence	Semi-structured individual interview	General	Cross-sectional: Not specified with respect to scans
Wernli et al., 2017 [32]	41	Breast	0–III	Post-treatment	Mammogram and breast MRI	Detect recurrence	Semi-structured interview in focus groups	Scan-specific	Cross-sectional: Not specified with respect to scans
Mixed methods studies									
Baun et al., 2020 [37]	38	Breast	IV	Living with cancer	CT or combined PET/CT	Detect progression/monitor treatment response	Quantitative assessment focused on patients’ use of electronic records for test results (not anxiety) followed by semi-structured individual interviews	General	Sequential: Not specified with respect to scans
Bellhouse et al., 2020 [41]	29	NSCLC	I-III	Living with cancer	Radiotherapy planning CT and two MRI scans approximately two weeks apart	Other: Radiotherapy planning	STAI-state Claustrophobia Questionnaire (CLQ) MRI anxiety questionnaire (MRI-AQ) CT anxiety questionnaire (CT-AQ) Semi-structured individual interview after last scan	General (STAI-S, CLQ) Scan-specific (MRI-AQ, CT-AQ, interview guide)	Longitudinal, concurrent: Baseline and after each MRI scan
Bui et al., 2022 [29]	222	Multiple; solid tumors	III, IV	Living with cancer	CT	Detect progression Investigative	Single item (experience scanxiety; yes/no) Modified Distress Thermometer Open-ended text response Other measures examined as correlates: six-item STAI, HADS, FOP-Q-SF	Scan-specific (single item, modified Distress Thermometer, open text) General (FOP, STAI, HADS)	Concurrent: Within four months after scan/scan results
Koo et al., 2017 [30]	12	Bladder	0; non-muscle-invasive	Living with cancer Post-treatment	Cystoscopy	Detect progression Detect recurrence	Psychological consequences of screening questionnaire (PCQ) Customer satisfaction survey (CSS) with items on worry and discomfort from procedure Semi-structured interview in focus groups	Scan-specific	Sequential: After cystoscopy procedure
Thompson, C.A. et al., 2010 [18]	70	Hodgkin’s lymphoma Aggressive non-Hodgkin’s lymphoma	Not specified	Post-treatment	CT	Detect recurrence	STAI-trait Semi-structured individual interviews	General (STAI) Scan-specific (interview guide)	Concurrent: Not specified with respect to scan (mean time since last scan = 14.8 months, SD 25.3)

**Table 4:** Quantitative measures of scanxiety used in included articles, according to scan-specific vs. general assessments.

Scan-Specific Measures (Worded with Respect to Scans)		General Measures (Worded without Reference to Scans)	
Measure	n, Articles	Measure	n, Articles
Impact of Events Scale (IES; revised or short form)	2 [3,17]	State-Trait Anxiety Inventory (STAI)	6 [13,18,20,41,48]
Modified Distress Thermometer	2 [29]	Fear of Cancer Recurrence Inventory (FCRI)	3 [27,28,43]
Psychological Consequences of Screening Questionnaire (PCQ)	2 [25,30]	Hospital Anxiety and Depression Scale (HADS)	3 [15,23,29]
MRI Anxiety Questionnaire	1 [41]	Cancer Worry Scale (CWS)	2 [14,24]
CT Anxiety Questionnaire	1 [41]	Claustrophobia Questionnaire	1 [41]
Medical Imaging Radiation Worry item from the HINTS survey	1 [38]	Breast Cancer Psychosocial Assessment	1 [42]
Stanford Acute Stress Reaction Questionnaire	1 [26]	Health Fears items	1 [22]
Customer Satisfaction Survey (worry and discomfort items)	1 [30]	SF-12 Quality of Life Mental Component Score	1 [16]
Study-specific item (anxiety during procedure, 10-point Likert)	1 [21]	Visual analog scale (VAS) items on fear of recurrence	1 [14]
Study-specific item (distress about components of follow-up, 4-point Likert)	1 [42]	Fear of Progression Questionnaire	1 [29]
Study-specific item (anxiety about diagnosis, not at all to completely)	1 [47]	PROMIS Global Anxiety	1 [27]
Study-specific item (anxiety during procedure, 5-point Likert)	1 [39]	Study-specific item, daily stress (0–10 Likert)	1 [25]
Study-specific item (anxiety about scan modalities, 5-point Likert)	1 [46]	Study-specific item, subjective anxiety (0–10 Likert)	1 [48]
Study-specific item (whether experienced scanxiety; yes/no)	1 [29]	Distress Thermometer (general)	1 [15]

Note: Measures were used to assess psychological symptoms around the time of scans or with consideration of scans in the study design. Scan-specific assessments were specifically worded in reference to a scan; general measures did not include a specific focus.

**Table 5:** Recommended areas for future scanxiety research.

***Research Topics: Expanding Research to Areas Needing Additional Attention***	
**Suggested Areas**	**Example Research Questions**
Investigate care partners’ scanxiety levels and supportive care needs	What are the scanxiety characteristics, levels, and support needs among care partners/family members? How do care partners’ scanxiety compare to and influence that of the cancer survivor?
Determine whether people with cancer experience similar anxiety and uncertainty while awaiting different types of tests	How does anxiety fluctuate in the time period around cancer-monitoring blood tests and results delivery? What are the components or dimensions of anxiety around these procedures? What are the correlates and effects of anxiety around other tests?
Explore how newer modes of scan results delivery (e.g., via electronic results release; via video or other remote clinical interactions) affect scanxiety	Which patients experience decreased anxiety when receiving faster, automated test results (e.g., in patient portals)—and which patients experience elevated anxiety? What support strategies may patients need in order to engage with and benefit from this format of results delivery?
Expand scanxiety research to under-investigated populations, time periods, and scan types	What are the scanxiety experiences and coping strategies of those with hematological malignancies? How does anxiety fluctuate between the scan procedure and the scan results? What factors may exacerbate or buffer against increases? How do patients cope with anxiety in the context of investigative scans prompted by new or worsening symptoms? What are the longitudinal patterns of scanxiety that occur over time with repeated scans?
Expand work on the effects of scanxiety and moderators of these effects	For whom and how does scanxiety affect one’s likelihood of adhering to follow-up care? How does scanxiety impact physical symptoms, communication in appointments, and other outcomes?
Determine intervention targets and test whether interventions are effective	Are brief interventions (e.g., just-in-time micro-interventions) acceptable, feasible, and efficacious for reducing anxiety at the time of scan procedures? Which coping strategies are most effective for managing uncertainty about what results may show? When is the optimal time to introduce a behavioral intervention with respect to scans?
***Research Methods: Strengthening how Scanxiety Studies Are Conducted***	
**Suggested Approaches**	**Example Research Questions/Directions**
Harmonize measures and examine psychometrics	How does scanxiety relate to close constructs such as fear of recurrence or progression, anticipatory anxiety, and state anxiety? Is it sufficient to use existing state anxiety measures to index scanxiety? Or are there advantages to developing specific measures that reflect multiple elements or specific components of scanxiety? Examine psychometrics of scanxiety measures
Improve description of follow-up care procedures	Strengthen descriptions of what typical follow-up procedures entail (e.g., exams, tests) In studies of general anxiety, include questions about whether an upcoming scan or scan discussion is occurring
Detail time periods to include information on procedures and scan results delivery phases	Describe the length of time between assessment time points with respect to pre-scan, post-scan, and results delivery time points. Report whether, when, and how results were delivered, and what they showed. How do waiting periods and results delivery methods influence scanxiety? How can clinicians or clinics structure the scan experience to help mitigate anxiety?
Explore innovative measurement strategies	Are daily diary and/or ecological momentary assessment approaches acceptable and feasible around the time of scans for older adults, those with advanced disease, and those on active treatment? Do these approaches reveal fluctuations and individual differences not evident from one-time assessments?
***Intervention Approaches: Developing and Testing Ways to Manage Scanxiety***	
**Promising Approaches**	**Example Research Directions/Intervention Targets**
Screening to identify those experiencing or at higher risk for scanxiety	Use scan-specific measures to identify those with scanxiety Prioritize at-risk individuals for interventions
Tailoring strategies to stressful time periods	Design interventions that address procedure-related and results-related components of scanxiety Optimize strategies for each time period (e.g., pre-scan; awaiting results)
Behavioral / self-management strategies	Promote self-efficacy for coping with progression/recurrence Bolster overall stress management skills (e.g., relaxation, pleasant activities) Facilitate just-in-time strategies for distinct periods (e.g., during or directly before scans)
Clinic or system strategies	Reduce scan-to-results waiting time Provide education/structure that promotes knowing what to expect for the procedure Engage in shared decision-making about scans and results delivery

## Supplementary Materials

The following supporting information can be downloaded at: https://www.mdpi.com/article/10.3390/cancers15051381/s1, File S1: OVID Search Strategy.

## References

[1] Feiler B. (2011). Scanxiety. Fear of a postcancer ritual. Time.

[2] Bui K.T., Blinman P., Kiely B.E., Brown C., Dhillon H.M. (2021). Experiences with scans and scanxiety in people with advanced cancer: A qualitative study. Support Care Cancer.

[3] Bauml J.M., Troxel A., Epperson C.N., Cohen R.B., Schmitz K., Stricker C., Shulman L.N., Bradbury A., Mao J.J., Langer C.J. (2016). Scan-associated distress in lung cancer: Quantifying the impact of “scanxiety”. Lung Cancer.

[4] Hurley-Browning L. (2018). What Is Scanxiety? Abramson Cancer Center—Penn Medicine. https://www.pennmedicine.org/cancer/about/focus-on-cancer/2018/october/what-is-scanxiety.

[5] Darisipudi S. Tools Patients, Survivors and Cancer Caregivers Use to Deal With ‘Scanxiety’. Cure Today. https://www.curetoday.com/view/tools-patients-survivors-and-cancer-caregivers-use-to-deal-with-scanxiety-.

[6] Peteet J.R., Stomper P.C., Ross D.M., Cotton V., Truesdell P., Moczynski W. (1992). Emotional support for patients with cancer who are undergoing CT: Semistructured interviews of patients at a cancer institute. Radiology.

[7] Mollica M.A., Smith A.W., Tonorezos E., Castro K., Filipski K.K., Guida J., Perna F., Green P., Jacobsen P.B., Mariotto A. (2022). Survivorship for Individuals Living With Advanced and Metastatic Cancers: National Cancer Institute Meeting Report. JNCI J. Natl. Cancer Inst..

[8] Bui K.T., Liang R., Kiely B.E., Brown C., Dhillon H.M., Blinman P. (2021). Scanxiety: A scoping review about scan-associated anxiety. BMJ Open.

[9] Munn Z., Peters M.D.J., Stern C., Tufanaru C., McArthur A., Aromataris E. (2018). Systematic review or scoping review? Guidance for authors when choosing between a systematic or scoping review approach. BMC Med. Res. Methodol..

[10] Tricco A.C., Lillie E., Zarin W., O’Brien K.K., Colquhoun H., Levac D., Moher D., Peters M.D.J., Horsley T., Weeks L. (2018). PRISMA Extension for Scoping Reviews (PRISMA-ScR): Checklist and Explanation. Ann. Intern. Med..

[11] Peters M.D.J., Marnie C., Tricco A.C., Pollock D., Munn Z., Alexander L., McInerney P., Godfrey C.M., Khalil H. (2020). Updated methodological guidance for the conduct of scoping reviews. JBI Evid. Synth..

[12] Derry-Vick H.M., Heathcote L.C., Stribling J., Glesby N., Luebke M., Epstein A.S., Prigerson H.G. (2021). Scan-Related Anxiety among Adults with Cancer: A Scoping Review Protocol. https://osf.io/b52y8/.

[13] Grilo A., Vieira L., Carolino E., Oliveira C., Pacheco C., Castro M., Alonso J. (2017). Anxiety in Cancer Patients during 18F-FDG PET/CT Low Dose: A Comparison of Anxiety Levels before and after Imaging Studies. Nurs. Res. Pract..

[14] McGinty H.L., Small B.J., Laronga C., Jacobsen P.B. (2016). Predictors and patterns of fear of cancer recurrence in breast cancer survivors. Health Psychol..

[15] Morreale M.K., Moore T.F., Kim S., Uphold H.S., Mabunda L.M., Harper F.W.K. (2020). Preferences for notification of imaging results in patients with metastatic cancer. Patient Educ. Couns..

[16] Patel H.D., Riffon M.F., Joice G.A., Johnson M.H., Chang P., Wagner A.A., McKiernan J.M., Trock B.J., Allaf M.E., Pierorazio P.M. (2016). A Prospective, Comparative Study of Quality of Life among Patients with Small Renal Masses Choosing Active Surveillance and Primary Intervention. J. Urol..

[17] Koinis F., Leontopoulou V., Chantzara E., Kotsakis A. (2022). “The Oizys Study”: Prevalence and impact of scan-related anxiety on QoL among Greek cancer patients and their caregivers. JCO.

[18] Thompson C.A., Charlson M.E., Schenkein E., Wells M.T., Furman R.R., Elstrom R., Ruan J., Martin P., Leonard J.P. (2010). Surveillance CT scans are a source of anxiety and fear of recurrence in long-term lymphoma survivors. Ann. Oncol..

[19] Wiedemann K., Smelser N.J., Baltes P.B. (2001). Anxiety and Anxiety Disorders. International Encyclopedia of the Social & Behavioral Sciences.

[20] Lo Re G., De Luca R., Muscarneri F., Dorangricchia P., Picone D., Vernuccio F., Salerno S., La Tona G., Pinto A., Midiri M. (2016). Relationship between anxiety level and radiological investigation. Comparison among different diagnostic imaging exams in a prospective single-center study. Radiol. Med..

[21] Abreu C., Grilo A., Lucena F., Carolino E. (2017). Oncological Patient Anxiety in Imaging Studies: The PET/CT Example. J. Cancer Educ..

[22] Cox C.L., Zhu L., Hudson M.M., Steen B.D., Robison L.L., Oeffinger K.C. (2013). Survivor typologies predict medical surveillance participation: The childhood cancer survivor study. Psycho-Oncol..

[23] Krajewski W., Koscielska-Kasprzak K., Rymaszewska J., Zdrojowy R. (2017). How different cystoscopy methods influence patient sexual satisfaction, anxiety, and depression levels: A randomized prospective trial. Qual. Life Res..

[24] McGinty H., Laronga C., Hicks C., Cases M., Rose M., Rodriguez Y., Jacobsen P. (2014). Sleep quality before and after surveillance mammograms in breast cancer survivors: Fear of cancer recurrence predicts longitudinal change in sleep quality. Psycho-Oncol..

[25] Porter L.S., Mishel M., Neelon V., Belyea M., Pisano E., Soo M.S. (2003). Cortisol levels and responses to mammography screening in breast cancer survivors: A pilot study. Psychosom. Med..

[26] Shelby R.A., Scipio C.D., Somers T.J., Soo M.S., Weinfurt K.P., Keefe F.J. (2012). Prospective Study of Factors Predicting Adherence to Surveillance Mammography in Women Treated for Breast Cancer. J. Clin. Oncol..

[27] Soriano E.C., Perndorfer C., Siegel S.D., Laurenceau J.P. (2019). Threat sensitivity and fear of cancer recurrence: A daily diary study of reactivity and recovery as patients and spouses face the first mammogram post-diagnosis. J. Psychosoc. Oncol..

[28] Soriano E.C., Perndorfer C., Otto A.K., Siegel S.D., Laurenceau J.P. (2018). Does sharing good news buffer fear of bad news? A daily diary study of fear of cancer recurrence in couples approaching the first mammogram post-diagnosis. Psycho-Oncol..

[29] Bui K.T., Kiely B.E., Dhillon H.M., Brown C., Xu K., Shafiei M., Blinman P. (2022). Prevalence and severity of scanxiety in people with advanced cancers: A multicentre survey. Support Care Cancer.

[30] Koo K., Zubkoff L., Sirovich B.E., Goodney P.P., Robertson D.J., Seigne J.D., Schroeck F.R. (2017). The Burden of Cystoscopic Bladder Cancer Surveillance: Anxiety, Discomfort, and Patient Preferences for Decision Making. Urology.

[31] Mannion S., Martin N.A., O’Connor J., Wieland J., Jatoi A. (2022). In Their Own Words, “Waiting Sucks:” A Qualitative Study of Medical Testing-Related Anxiety in Patients with Cancer. Am. J. Hosp. Palliat. Care.

[32] Wernli K., Brandzel S., Buist D., Bush M., DeMartini W., Ichikawa L., Haas C., Henderson L., Johnson D., Kerlikowske K. (2019). Is Breast MRI Better at Finding Second Breast Cancers than Mammograms Alone for Breast Cancer Survivors?. Patient Cent. Outcomes Res. Inst..

[33] Pascal J., Endacott R. (2010). Ethical and existential challenges associated with a cancer diagnosis. J. Med. Ethics.

[34] Lai-Kwon J., Heynemann S., Flore J., Dhillon H., Duffy M., Burke J., Briggs L., Leigh L., Mileshkin L., Solomon B. (2021). Living with and beyond metastatic non-small cell lung cancer: The survivorship experience for people treated with immunotherapy or targeted therapy. J. Cancer Surviv..

[35] Derry-Vick H., Hahne J., Saxena A., Glesby N., Epstein A., Lichtenthal W.G., Prigerson H. (2022). Mixed methods study to inform adaptation of a stress management intervention for advanced cancer patients awaiting scan results. Ann. Behav. Med..

[36] Sterba K.R., Zapka J., LaPelle N., Armeson K., Ford M.E. (2015). A Formative Study of Colon Cancer Surveillance Care: Implications for Survivor-Centered Interventions. J. Cancer Educ..

[37] Baun C., Vogsen M., Nielsen M.K., Hoilund-Carlsen P.F., Hildebrandt M.G. (2020). Perspective of Patients with Metastatic Breast Cancer on Electronic Access to Scan Results: Mixed-Methods Study. J. Med. Internet Res..

[38] Hay J.L., Baser R.E., Westerman J.S., Ford J.S. (2018). Prevalence and Correlates of Worry About Medical Imaging Radiation Among United States Cancer Survivors. Int. J. Behav. Med..

[39] Goense L., Borggreve A.S., Heethuis S.E., van Lier A.L., van Hillegersberg R., Mook S., Meijer G.J., van Rossum P.S.N., Ruurda J.P. (2018). Patient perspectives on repeated MRI and PET/CT examinations during neoadjuvant treatment of esophageal cancer. Br. J. Radiol..

[40] Brandzel S., Rosenberg D.E., Johnson D., Bush M., Kerlikowske K., Onega T., Henderson L., Nekhlyudov L., DeMartini W., Wernli K.J. (2017). Women’s experiences and preferences regarding breast imaging after completing breast cancer treatment. Patient Prefer. Adherence.

[41] Bellhouse S., Brown S., Dubec M., Taylor S., Hales R., Whiteside L., Yorke J., Faivre-Finn C. (2020). Introducing magnetic resonance imaging into the lung cancer radiotherapy workflow—An assessment of patient experience. Radiography.

[42] Bjelic-Radisic V., Dorfer M., Tamussino K., Greimel E. (2017). Patients’ view of routine follow-up after breast cancer treatment. Wien. Klin. Wochenschr..

[43] Jeppesen M.M., Jensen P.T., Hansen D.G., Christensen R.D., Mogensen O. (2018). Patient-initiated follow up affects fear of recurrence and healthcare use: A randomised trial in early-stage endometrial cancer. BJOG: Int. J. Obstet. Gynaecol..

[44] Allen A. (2002). The meaning of the breast cancer follow-up experience for the women who attend. Eur. J. Oncol. Nurs..

[45] Thompson H.S., Littles M., Jacob S., Coker C. (2006). Cancer survivors of African descent posttreatment breast cancer surveillance and follow-up care experiences of breast—An exploratory qualitative study. Cancer Nurs..

[46] Westerterp M., van Westreenen H.L., Deutekom M., Stoker J., Fockens P., Comans E.F., Plukker J.T., Bossuyt P.M., van Lanschot J.J., Sloof G.W. (2008). Patients’ perception of diagnostic tests in the preoperative assessment of esophageal cancer. Patient Prefer. Adherence.

[47] Derry H.M., Maciejewski P.K., Epstein A.S., Shah M.A., LeBlanc T.W., Reyna V., Prigerson H.G. (2019). Associations between Anxiety, Poor Prognosis, and Accurate Understanding of Scan Results among Advanced Cancer Patients. J. Palliat. Med..

[48] Martinez-Lorca A. (2022). Influence of Music in Anxiety Reduction in 18F-FDG PET-CT Studies. Int. J. Radiol. Imaging Technol..

[49] Sklenarova H., Krümpelmann A., Haun M.W., Friederich H.-C., Huber J., Thomas M., Winkler E.C., Herzog W., Hartmann M. (2015). When do we need to care about the caregiver? Supportive care needs, anxiety, and depression among informal caregivers of patients with cancer and cancer survivors. Cancer.

[50] Trevino K.M., Prigerson H.G., Maciejewski P.K. (2018). Advanced cancer caregiving as a risk for major depressive episodes and generalized anxiety disorder. Psycho-Oncol..

[51] Hahne J., Carpenter B., Epstein A., Prigerson H.G., Derry-Vick H.M. (2022). Communication skills training for oncology clinicians after the 21st Century Cures Act: The need to contextualize patient portal-delivered test results. JCO Oncol. Pract..

[52] Heathcote L.C., Cunningham S.J., Webster S.N., Tanna V., Mattke E., Loecher N., Spunt S.L., Simon P., Dahl G., Walentynowicz M. (2022). Smartphone-based Ecological Momentary Assessment to study “scanxiety” among Adolescent and Young Adult survivors of childhood cancer: A feasibility study. Psychooncology.

[53] Rankin K., Walsh L.C., Sweeny K. (2019). A better distraction: Exploring the benefits of flow during uncertain waiting periods. Emotion.

[54] Johnson J.A., Zawadzki M.J., Materia F.T., White A.C., Smyth J.M. (2022). Efficacy and acceptability of digital stress management micro-interventions. Procedia Comput. Sci..

