# Supplementary material for: Scanxiety among Adults with Cancer: A Scoping Review to Guide Research and Interventions
Source: Cancers (Basel). 2023 Feb 22;15(5):1381. doi: 10.3390/cancers15051381 (PMC10000102; doi:10.3390/cancers15051381)
Supplement: Supplementary file 1 [file cancers-15-01381-s001.zip › cancers-2176424-supplementary.pdf]

Supplementary Material

Ovid Search Strategy

1	exp Cancer survivors/px
2	cancer survivor*.mp
3	((long term or long-term) and cancer survivor*).mp
4	(survivor* and (cancer* or neoplasm*)).mp
5	Exp Neoplasms/dg, rt, rh [Diagnostic imaging, radiotherapy, rehabilitation]
6	(cancer* or neoplasm*).mp
7	(malignanc* or malignant neoplasm*).mp
8	neoplasia*.mp
9	tumor*.mp
10	carcino*.mp
11	oncol*.mp
12	1 or 2 or 3 or 4 or 5 or 6 or 7 or 8 or 9 or 10 or 11
13	exp magnetic resonance imaging/ or exp positron emission tomography computed tomography/ or exp tomography, optical coherence/ or exp tomography, x-ray/ or exp tomography, x-ray computed/ or exp single photon emission computed tomography computed tomography/ or exp tomography, spiral computed/ or exp multidetector computed tomography/ or exp spiral cone-beam computed tomography/ or exp x-ray microtomography/ or exp ultrasonography
14	chemical shift imaging*.mp
15	(echo adj3 spin).mp
16	functional mri*.mp
17	functional magnetic resonance imaging.mp
18	tomography.mp
19	(imaging* adj3 chemical shift).mp
20	(imaging and (magnetic resonance or nmr or spin)).mp

21	magnetic resonance imaging.mp
22	(tomography and (mr or nmr or proton)).mp
23	spin echo imaging*.mp
24	(scan* and mri).mp
25	exp follow-up studies/or exp longitudinal studies/or exp prospective studies/ or exp retrospective studies/ or exp controlled before-after studies/ or exp sampling studies/
26	((follow up or follow-up or followup) and stud*).mp
27	exp mammography/ or exp xeromammography/ or exp radiographic magnification/ or exp radiography, abdominal/ or exp spectroscopy, near infrared/
28	(digital adj3 tomosynthes*).mp
29	digital mammograph*.mp
30	(3d-mammograph* or 3d mammograph*).mp
31	(breast adj3 (digital or x-ray)).mp
32	(mammograph* and digital).mp
33	exp diagnostic imaging/ or exp image interpretation, computer-assisted/or exp neuronavigation/or exp radiographic image interpretation, computer-assisted/or exp tomography, emission-computed/ or exp positron-emission tomography/ or exp tomography, emission-computed, single-photon/ or exp tomography, x-ray computed/ or exp imaging, three-dimensional/ or exp microscopy/ or exp radiography
34	diagnostic imaging*.mp
35	(imaging and (diagnostic or medical)).mp
36	Medical imaging.mp
37	exp x-rays/
38	(x and (radiation* or ray*)).mp
39	X ray.mp
40	13 or 14 or 15 or 16 or 17 or 18 or 19 or 20 or 21 or 22 or 23 or 24 or 25 or 26 or 27 or 28 or 29 or 30 or 31 or 32 or 33 or 34 or 35 or 36 or 37 or 38 or 39
41	anxiety/ or anxiety.mp
42	depression.mp or exp depression/

43	*anxiety/ or exp psychological distress/
44	anxiet*.mp
45	anxieties social.mp
46	social anxiet*.mp
47	(nervousness or hypervigilance).mp
48	anxiety disorders/dg, et, px [diagnostic imaging, etiology, psychology]
49	(anxiety and (disorder* or neuroses)).mp
50	(anxiety adj3 neurotic).mp
51	disorder* anxiety.mp
52	neuroses anxiety.mp
53	neurotic anxiety state*.mp
54	(state adj3 anxiety).mp
55	depression.mp. or exp depression/
56	depression*.mp
57	depression* emotional.mp
58	(depressive symptom* or emotional depression*).mp
59	symptom* depressive*.mp
60	depressive disorder.mp or exp depressive disorder/
61	(depression and (endogenous or neurotic or syndrome)).mp
62	(depressive and (disorder* or neuro* or syndrome*)).mp
63	fear.mp or exp fear/
64	fears.mp
65	stress psychological.mp. or exp stress, psychological/
66	life stress*.mp
67	mental suffering.mp
68	psychologic* stress*.mp
69	(stress* and (life or psycholog*)).mp

70	suffering*.mp
71	41 or 42 or 43 or 44 or 45 or 46 or 47 or 48 or 49 or 50 or 51 or 52 or 53 or 54 or 55 or 56 or 57 or 58 or 59 or 60 or 61 or 62 or 63 or 64 or 65 or 66 or 67 or 68 or 69 or 70
72	12 and 40 and 71
